# Risk Factors and Predictive Biomarkers for Postoperative Complications in Crohn’s Disease Surgery: Systematic Review

**DOI:** 10.3390/ijms27135731

**Published:** 2026-06-25

**Authors:** Bobuțac Eduard, Zaharie Delia Roxana, Vălean Dan, Emil Moiș, Călin Popa, Andra Ciocan, Nadim Al-Hajjar, Florin Zaharie

**Affiliations:** 1 Department of Surgery, University of Medicine and Pharmacy “Iuliu Hațieganu”, 400012 Cluj-Napoca, Romania; eduard.bobutac@gmail.com (B.E.); valean.d92@gmail.com (V.D.); drmoisemil@gmail.com (E.M.); calinp2003@yahoo.com (C.P.); andra.ciocan10@gmail.com (A.C.); na_hajjar@yahoo.com (N.A.-H.); florinzaharie@yahoo.com (F.Z.); 2 Regional Institute of Gastroenterology and Hepatology “Octavian Fodor”, 400162 Cluj-Napoca, Romania

**Keywords:** Crohn’s disease, postoperative complications, biomarkers, neutrophile-to-lymphocyte ratio, C-reactive protein, sarcopenia, machine learning

## Abstract

Surgical intervention in Crohn’s disease remains a significant contributor to patient morbidity, with postoperative complication rates reported between 20% and 50%. These complications include a broad spectrum of adverse outcomes, such as surgical site infections, intra-abdominal abscesses, and anastomotic leakage, all of which can substantially impact recovery, healthcare costs, and long-term prognosis. Although several clinical and perioperative risk factors have been identified, accurate prediction of postoperative outcomes remains challenging, highlighting the need for improved risk stratification strategies. In recent years, the evolution of biological therapies has transformed the management of Crohn’s disease, raising important questions regarding their influence on surgical outcomes and postoperative healing. Consequently, a more nuanced understanding of the interplay between medical and surgical approaches is required to optimize patient care. This systematic review aims to evaluate established and emerging predictive biomarkers associated with postoperative complications in Crohn’s disease surgery. Particular emphasis is placed on inflammatory markers, nutritional parameters, and novel molecular signatures. Furthermore, the review explores the growing role of multiomics approaches—including genomics, proteomics, and metabolomics—as well as the integration of machine learning models to enhance predictive accuracy. By synthesizing current evidence, this study underscores the potential of combining biomarkers with advanced analytical tools to support personalized risk assessment and guide clinical decision-making in Crohn’s disease surgery.

## 1. Introduction

Crohn’s disease (CD) is a chronic inflammatory bowel disease characterized by a relapsing–remitting course, with alternating periods of recurrence and remission that lack a clearly defined pattern and are often unpredictable [1]. The disease may involve any segment of the gastrointestinal tract; however, most patients present with involvement of the terminal ileum and proximal colon [2]. Initially, the disease course is marked by episodes of remission and exacerbation accompanied by a characteristic set of symptoms. As the disease progresses, however, permanent morphological changes in the intestine develop, leading to irreversible intestinal dysfunction [3] and the emergence of complications requiring surgical intervention. According to available studies, approximately 40% of patients diagnosed with CD develop surgical complications within 20 years of diagnosis [1,2,3,4], and one-third of these patients will require at least one additional resection surgery within the subsequent 10 postoperative years [5].

Moreover, following ileocolic resection, endoscopic recurrence has been reported in up to 73% of patients within the first year after surgery [3,6]. The need for repeat surgical intervention within the first 10 years after the initial operation ranges between 31% and 50% [6,7]. A population-based cohort study in adult patients with Crohn’s disease (CD) reported cumulative probabilities for major abdominal surgery of 38% at 5 years, 48% at 10 years, and 58% at 20 years following diagnosis [8].

Surgical complications include fibrotic strictures leading to bowel obstruction or subocclusive syndromes, intestinal perforation, enteric or colonic fistulas, gastrointestinal hemorrhage, intra-abdominal abscess formation, and malignant transformation [9]. These complications are often exacerbated by the patients’ poor preoperative condition, largely attributable to the underlying disease. The identification of reliable biomarkers capable of accurately reflecting the inflammatory status in these patients would facilitate risk stratification and enable more effective optimization of both disease progression and preoperative status [10,11]. Studies focusing on biomarkers, including those employing computational modeling and artificial intelligence for data analysis, have demonstrated a higher incidence of surgical complications and postoperative recurrence in CD patients with abnormal levels of albumin [12], C-reactive protein [13], and hemoglobin [12,13,14]. In contrast, biomarkers such as leukocyte count, platelet count, urea, and creatinine have not shown consistent clinical relevance. Genetic variants of specific genes, as well as genomic biomarkers, also play a significant role and may be utilized to predict the risk of surgical complications in patients with CD [15].

The purpose of this review is to highlight and synthesize the current evidence regarding potential biomarkers with a predictive role in postoperative complications in patients with Crohn’s disease. By identifying clinically relevant inflammatory, biochemical, and genomic markers, this study aims to contribute to improved risk stratification, perioperative optimization, and ultimately better surgical outcomes in this patient population. The primary objective of this systematic review is to synthesize and evaluate contemporary evidence regarding the clinical, biological, and technological predictors of postoperative complications in patients undergoing surgery for Crohn’s disease. By critically appraising established inflammatory biomarkers, nutritional indices, and emerging machine learning algorithms within the 2015–2026 framework, this review aims to provide an evidence-based perspective on perioperative risk stratification. The ultimate goal is to identify reliable markers that can inform personalized optimization strategies and improve surgical recovery outcomes in this complex and high-risk patient population.

## 2. Materials and Methods

**Figure 1:**
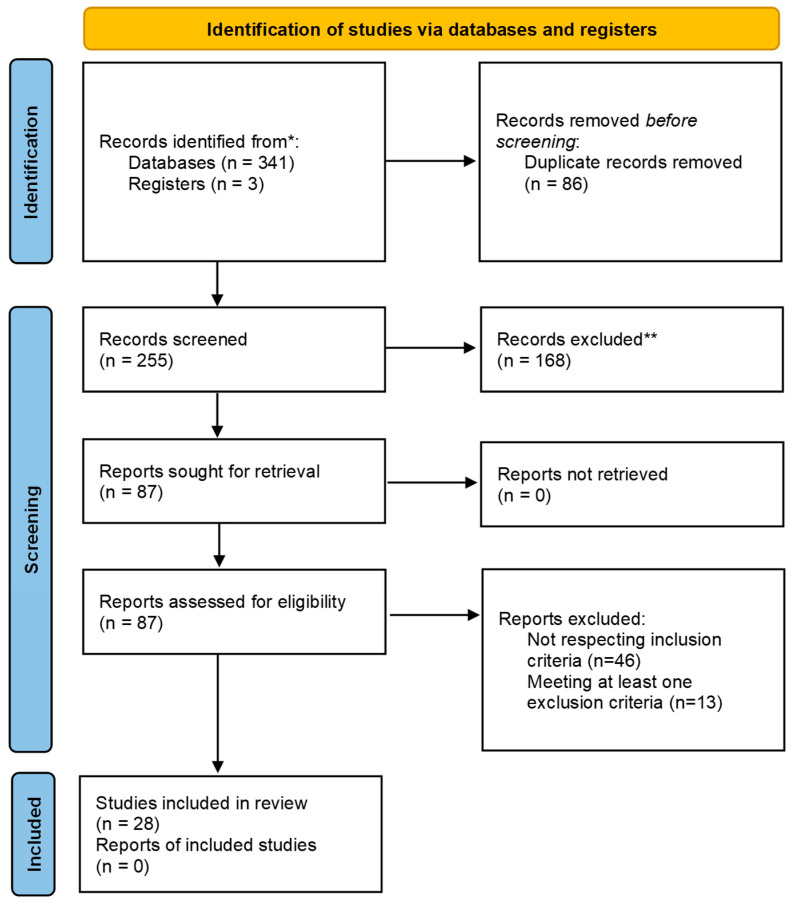
Prisma Flowchart for selected studies. * Records respecting the primary inclusion criteria. ** Records who fit the exclusion criteria.

This systematic review was conducted in accordance with the PRISMA 2020 (Preferred Reporting Items for Systematic Reviews and Meta-Analyses) guidelines. The study selection process, including identification, screening, eligibility, and inclusion, was documented using a PRISMA flow diagram (Figure 1) [16].

### 2.1. Search Strategy

A comprehensive literature search was performed across three major electronic databases: PubMed, Embase, and Scopus. The search covered studies published between January 2015 and April 2026.

The research was performed according to the Preferred Reporting Items for a Systematic Review and Meta-Analysis of Individual Participant Data Criteria. The search strategy was based on the following primary keywords: “Crohn’s disease surgery”, “postoperative complications”, “prediction”, and “biomarkers”. These terms were used both individually and in combination, employing Boolean operators (AND, OR) to maximize sensitivity and identify all potentially relevant studies.

To address the requirement for methodological transparency, the research objective was formalized using the PICO (Population, Intervention, Comparator, and Outcome) framework [17]:

Population (P): Adult patients (age more than 18) with an established diagnosis of Crohn’s disease undergoing any form of intestinal resection or strictureplasty.

Intervention/Exposure (I): Preoperative and intraoperative risk factors (e.g., biological therapy, malnutrition, smoking, disease phenotype, or surgical technique).

Comparator (C): Patients undergoing the same surgical procedures without the specific risk factor or exposure.

Outcome (O): Primary outcomes included overall postoperative complications (within 30 days) and Intra-Abdominal Septic Complications (IASCs), such as anastomotic leaks, abscesses, or enteric fistulas. Secondary outcomes included surgical recurrence and length of hospital stay (LOHS).

The search primarily focused on identifying clinical trials, retrospective cohort studies, and key systematic reviews, evaluating predictive biomarkers for postoperative complications. The selection process was divided into two stages: the primary stage was focused on reading the abstracts and titles, and the secondary stage was focused on analyzing the contents for further review. In addition, selection criteria were performed within respect to these articles.

Studies were considered eligible if they met the following inclusion criteria:

1. Involved adult patients diagnosed with Crohn’s disease.
2. Evaluated postoperative complications following surgical intervention.
3. Investigated the predictive role of inflammatory, biochemical, or genetic biomarkers.
4. Were original research articles published in peer-reviewed journals.
5. Were available in English.

Exclusion criteria included:

1. Case reports, editorials, letters to the editor;
2. Studies involving pediatric populations only;
3. Articles lacking sufficient data on postoperative outcomes or biomarker analysis;
4. Duplicate publications or overlapping datasets.

### 2.2. Study Selection

A total of 341 articles were initially identified based on relevance to the research topic. After removal of duplicates and preliminary screening of titles and abstracts, full-text evaluation was performed. Following a thorough review and critical appraisal of the selected studies, 28 articles met the predefined eligibility criteria and were included in the final analysis.

### 2.3. Data Extraction and Synthesis

Data extraction was performed systematically, focusing on study characteristics (author, year, study design), patient population, type of surgical intervention, biomarkers assessed, and reported postoperative outcomes. Emphasis was placed on identifying biomarkers with potential predictive value for postoperative complications. Given the heterogeneity of study designs, populations, and outcome measures, a qualitative synthesis of the findings was performed rather than a meta-analysis. Table 1 highlights the selected articles for review.

### 2.4. Risk of Bias Assessment

The methodological quality of the included studies was assessed through critical appraisal of study design, sample size, selection criteria, and reporting of outcomes. Potential sources of bias, including selection bias, reporting bias, and confounding factors, were considered during data interpretation.

The methodological quality of the observational studies included was independently assessed by two reviewers using the Newcastle–Ottawa Scale (NOS). Studies were evaluated across three core domains: (1) selection of the study groups (0–4 points), (2) comparability of the groups based on the design or analysis (0–2 points), and (3) outcome assessment (0–3 points). For the comparability domain, we prioritized studies that controlled critical confounders in Crohn’s disease surgery. Studies achieving a total score of over 7 stars were categorized as “Good” while scores of 5–6 and less than 5 were considered “Fair” and “Low” respectively. Any discrepancies in scoring were resolved through discussion or consultation with a third senior reviewer. Furthermore, the certainty of the body of evidence was evaluated using the Grading of Recommendations Assessment, Development and Evaluation (GRADE) framework, which classifies evidence as high, moderate, low, or very low based on risk of bias, inconsistency, indirectness, and imprecision.

## 3. Results and Discussion

The quality assessment of the included studies, grouping based on their conclusions and key findings, and the risk of bias are highlighted in Table 2. When evaluated with the Newcastle–Ottawa scale, 26/28 articles received a “Good” mark, one article was considered of “Fair” grading, whereas one article could not be marked due to its editorial state.

To move past a purely descriptive analysis of individual laboratory values, a systematic stratification of these markers based on their clinical domain and pathophysiological mechanisms is required. By categorizing these predictors into nutritional/morphometric, systemic inflammatory, local inflammatory, and advanced computational archetypes, clinical teams can more effectively isolate distinct pathways of perioperative risk. Furthermore, evaluating these parameters through the standardized Grading of Recommendations Assessment, Development and Evaluation (GRADE) framework reveals a clear disparity between traditional biochemistry and cutting-edge analytical tools. While basic nutritional and acute-phase reactants possess a high certainty of evidence backed by massive population databases, newer technological metrics face widespread heterogeneity and structural biases that limit immediate, standalone utility. A comprehensive synthesis of these biomarker categories, including their graded strength of evidence, verified clinical cut-offs, and immediate perioperative management implications, is systematically detailed in Table 3.

### 3.1. General Considerations

The surgical landscape for Crohn’s disease (CD) has undergone a paradigm shift within the 2015–2026 framework, transitioning from reactive intervention to a sophisticated model of perioperative optimization and risk stratification. Despite the advent of potent biological therapies and small molecules that have reduced the overall incidence of major abdominal surgery, nearly one-third of patients still require surgical resection within five years of diagnosis [47]. Surgery in this population is inherently high-risk, with postoperative complication rates remaining between 20% and 50% [48,49]. Identifying reliable predictive factors and biomarkers is therefore a clinical necessity to improve patient outcomes and mitigate the substantial healthcare burden associated with CD surgery.

The initial assessment of surgical risk in Crohn’s disease relies heavily on the clinical phenotype and history of the patient. The Montreal classification provides a foundational layer for this stratification, identifying disease behavior and location as critical determinants of postoperative outcomes [50,51]. Within contemporary literature, penetrating disease behavior (B3) consistently emerges as a significant risk factor for infectious complications, such as Intra-Abdominal Septic Complications (IASCs) and anastomotic leakage. This behavior is characterized by the formation of fistulae and abscesses, creating a “hostile” surgical environment that complicates both the resection and the subsequent healing of the anastomosis. In a cohort analysis by Uchino et al. [52] evaluating the risk of preoperative biological therapy used, the primary risk factors for postoperative complications were the current smoking status (OR = 3.44), emergency surgery (OR = 6.85) and abdominoperineal resection (OR = 14.93). Notably, penetrating disease (OR = 14.55), smoking status (OR = 7.09) and ASA greater than 3 (OR = 5.85) were identified as risk factors for incisional SSI [52]. Emerging data also highlight the importance of non-modifiable factors such as patient sex and age. While older age is a well-established risk factor for general surgical morbidity due to frailty and comorbidities, younger age at first resection (specifically less than 28 years) has been identified as a potent predictor of surgical recurrence (HR = 16.44), possibly reflecting a more aggressive disease biology in early-onset cases [53]. Interestingly, recent studies have noted that men may experience significantly more complications than women (*p* = 0.018), suggesting potential hormonal or behavioral influences on surgical recovery. These findings are also supported by Kawamoto et al. [22], who, over a cohort of 206 patients, highlighted that increased duration of surgery (over 173 min), as well as penetrating disease, can be independent risk factors for SSIs [22]. Naveh et al. [20] highlighted a higher rate of complications in patients with a longer interval from diagnosis to surgery [20].

### 3.2. Inflammatory Markers: Hematological and Localized Insights

The shift toward objective biomarkers has allowed for more granular risk stratification. Routine hematological parameters, which reflect the balance between innate and adaptive immunity, have gained prominence as cost-effective predictive tools. Beyond traditional markers, the integration of hematological ratios like the neutrophil-to-lymphocyte ratio (NLR) and platelet-to-lymphocyte ratio (PLR) provides a nuanced window into the “hostile” immunological environment of Crohn’s disease (CD). An elevated PLR reflects a state of systemic inflammation where interleukin-6 drives thrombocytosis, while simultaneously, chronic inflammation exhausts the lymphocyte pool. This imbalance is not merely a marker of disease activity but a functional predictor of poor healing [54]. Research by Kawamoto et al. [22] identified a preoperative neutrophil-to-lymphocyte ratio NLR < 3.89 as an independent predictor of IASC (OR = 3.43, *p* = 0.013), noting that the NLR may reflect intestinal-specific inflammatory activity that does not always manifest as changes in C-reactive protein (CRP) [22]. This is reinforced by Mullin et al. [31] and colleagues, who conducted an 11-year retrospective study involving patients with CD who underwent surgery for disease-related complications within a single medical center. The authors investigated the association between several inflammatory markers—including platelet-to-lymphocyte ratio (PLR), neutrophil-to-lymphocyte ratio (NLR), lymphocyte-to-monocyte ratio (LMR), and the modified systemic inflammatory score (mSIS)—and their prognostic value in surgically treated CD patients. Within the same study, they also evaluated the relationship between PLR and major postoperative complications. Elevated PLR was associated with increased rates of major postoperative complications and higher reintervention rates in elective procedures [31]. This association is biologically plausible, as thrombocytosis reflects systemic inflammation, with platelet counts increasing under the influence of interleukin-6, while lymphocyte levels tend to decrease inversely with inflammatory activity. Consequently, an elevated PLR may serve as an indicator of severe inflammation and could be considered a useful prognostic tool not only in CD but also in other surgical pathologies [55]. The lymphocyte-to-monocyte ratio (LMR) has also been investigated in relation to inflammatory disease progression. Higher LMR values have been associated with improved survival outcomes in inflammatory and infectious conditions, suggesting a potential protective or prognostic role.

Mullin et al.’s findings demonstrated that elevated NLR was associated with an increased risk of major postoperative complications requiring reintervention in elective surgeries, as well as a higher incidence of thromboembolic events [31]. Additionally, increased NLR values were correlated with disease recurrence. The authors further linked elevated NLR to an increased risk of thrombosis and postoperative complications, supported by findings from other studies, such as those by Kang et al., which reported a 2.78-fold higher risk of complications in patients with NLR ≥ 4.1 [56]. Nevertheless, conflicting evidence exists. For instance, Argeny et al. reported in a cohort of 373 patients who underwent CD surgery, lower NLR values in patients who developed complications (3.77 vs. 4.67, *p* = 0.001), despite the presence of penetrating disease or malignancy, highlighting the heterogeneity of findings across studies, however linking the marker to specific disease phenotypes such as inflammatory masses, abscesses or malignancy in the resected specimen, thus prompting the need for further studies [41].

While hematological ratios offer systemic insights, C-reactive protein remains the “gold standard” for assessing acute inflammation. Teixeira et al. [19] reported that elevated preoperative CRP was the only independent predictor of 30-day adverse outcomes (OR = 1.31, *p* = 0.049) in a multicenter cohort of 79 patients, highlighting the importance of perioperative management. Furthermore, localized inflammation at the resection site is increasingly monitored via fecal calprotectin (FCP) [19]. Naveh et al. [20] found that preoperative FCP was the only factor significantly influencing the severity of complications as measured by the Clavien–Dindo grade (*p* = 0.05). In a cohort of 242 patients, they concluded that higher FCP levels correlate with a more difficult recovery, as active mucosal inflammation predisposes to anastomotic dehiscence and localized abscess formation. Moreover, they highlighted that postoperative recurrence is less likely to be determined by surgical or perioperative factors [20] This is also supported by Saleh et al. [21].

Studies investigating inflammatory biomarkers and their predictive value for surgical complications in CD suggest an increased incidence of postoperative complications in patients with low hemoglobin, low albumin, and elevated C-reactive protein (CRP) levels. Ahmed S. Ghoneima and colleagues conducted a 5-year retrospective study including 79 patients (24 men and 55 women) who underwent laparoscopic intestinal resection. The study demonstrated that abnormal levels of hemoglobin, albumin, and CRP were associated with a higher frequency of postoperative complications in CD. However, the study excluded patients undergoing open, robotic, or hand-assisted procedures, as well as emergency surgeries or operations without intestinal resection [37].

Given the relatively small sample size and the exclusion of a substantial number of patients, as well as the focus on septic complications, these findings should be interpreted with caution and may not be generalizable to all CD patients with surgical complications. A separate study by Stidham et al., focusing on postoperative infectious complications, evaluated biological parameters measured within 48 h prior to surgery in 269 patients undergoing operative treatment for CD complications. The authors concluded that low hemoglobin levels (11.96 vs. 11.13 g/dL, *p* < 0.001) were a significant predictor of complications requiring surgical management. Other parameters, including leukocyte count, platelet count, urea, and creatinine, were not found to be statistically significant predictors. It should be noted that patients presenting with intra-abdominal abscesses or peritoneal perforation were excluded from this study, which may influence the overall complication rates reported in the context of our review [45]. Furthermore, Hu et al. [40] highlighted over a cohort study of 159 patients, that anemia (OR = 3.31, *p* = 0.04), neutrophilia (OR = 2.85, *p* = 0.014), and intraoperative findings of fistula (OR = 3.76, *p* = 0.004), were independent predictors in developing postoperative SSI, suggesting that favorable prenutrition status as well as a low inflammatory status may lower the postoperative SSI incidence [40]. In a similar manner of evaluating systemic inflammatory equilibrium, Zhao et al. [32] identified, over a cohort of 534 patients, the postoperative C-reactive protein-to-albumin ratio (CAR) as a formidable independent predictive marker. Utilizing a specific cutoff threshold of 3.25 on postoperative day 3, CAR demonstrated significantly higher accuracy in forecasting surgical site infections and prolonged hospital stays than standalone traditional CRP tracking [32].

To encapsulate these multi-system deficits, Dong et al. [35] validated the Controlling Nutritional Status (CONUT) score on a cohort of 202 patients, demonstrating that a preoperative CONUT score higher than 3.5—which merges serum albumin, total cholesterol, and peripheral lymphocyte counts—acts as a robust predictor of complications. This compound immuno-nutritional score ultimately outperforms single-variable metrics by capturing simultaneous nutritional and immunological exhaustion. Postoperative complications were correlated with BMI, preoperative albumin, the preoperative CONUT score, and preoperative infliximab use [35].

### 3.3. Role of Nutrition and Morphomics in Predicting Postoperative Complications

Malnutrition is one of the most potent modifiable risk factors for surgical complications in Crohn’s disease. Traditional measures like Body Mass Index (BMI) are increasingly viewed as insufficient, as they fail to capture the distribution of lean muscle versus fat. A large-scale National Surgical Quality Improvement Program (NSQIP) study by Nguyen et al. [38] included 10,913 patients from the United States and Canada (6082 with Crohn’s disease and 4831 with ulcerative colitis). The results demonstrated significantly higher mortality rates in patients with moderate and severe hypoalbuminemia (0.7% vs. 0.2%, *p* < 0.05; 2.4% vs. 0.2%, *p* < 0.01) [38]. Additionally, these patients exhibited increased rates of intra-abdominal infectious complications, sepsis, pneumonia, and non-septic extraintestinal complications such as wound dehiscence and postoperative bleeding compared to patients with normal albumin levels. Of relevance to the present review, patients with severe hypoalbuminemia showed a higher rate of reoperation for major surgical intervention within 30 days of the initial procedure (8.7% vs. 6.3%, *p* < 0.01). These findings are consistent with other studies reporting increased reintervention rates in hypoalbuminemic patients undergoing total abdominal colectomy with terminal ileostomy. Therefore, severe hypoalbuminemia may serve as a marker of advanced disease severity and is associated with poorer postoperative outcomes, supporting its role as an indirect indicator of prognosis and systemic complication risk.

Stidham et al. [45] also refined this statement by using “analytic morphomics,” concluding that subcutaneous-to-visceral fat distribution was a superior predictor of postoperative complications as well as reduced levels of albumin. Low albumin levels should be carefully considered in patients with CD, as hypoalbuminemia is identified in approximately 25–50% of hospitalized patients with this condition [32]. Multiple studies have demonstrated an increased rate of both preoperative and postoperative complications in patients with hypoalbuminemia. This condition is frequently associated with malnutrition, active inflammation, and sepsis, and preoperative hypoalbuminemia is widely regarded as a strong predictor of postoperative morbidity and mortality. Thus, preoperative nutritional index (PNI) can play an important role in the minimization of postoperative complications, as highlighted by Bae et al. [46] in 2024. Over a cohort of 227 patients divided into two groups (low PNI vs. high PNI), the low PNI group had significantly higher rates of postoperative complications (32% vs. 14%, *p* = 0.001); in addition, a low PNI as well as longer operation time were highlighted as independent predictors of postoperative infectious complications [46]. Furthermore, in a study published by Jiang et al. [27], in a cohort of 98 patients, preoperative enteral nutrition yielded lower postoperative complication rates, highlighting a significant decrease in the need for reintervention, particularly mitigating the risk of prolonged ileus linked to malnutrition [27].

Modern assessment focuses on sarcopenia, which is an important component of the frailty index of the patients. Saleh et al. [21] published a systematic review, pooling 14 studies consisting of a total of 2334 patients, demonstrating that sarcopenia increases the risk of developing abscesses 5.03-fold (*p* = 0.0004). Sarcopenia was also associated with a higher risk of hospitalization (OR = 1.87); however, it presented no statistically significant difference compared to non-sarcopenic patients regarding postoperative anastomotic leaks [21]. Moreover, Zhang et al. [33] similarly identified in a cohort of 134 patients that sarcopenia is an independent risk factor for major postoperative complications (OR = 3.97, *p* = 0.027), noting it often co-exists with low preoperative albumin and hemoglobin [33]. It is worth noting that sarcopenic patients were also associated with longer hospitalization, more occurrences of complications, most notably infection and intestinal fistula, which is in contradiction with Saleh et al.

A significant portion of the newly added literature underscores the critical role of preoperative nutritional depletion and altered body composition as independent drivers of adverse surgical outcomes in Crohn’s disease patients. Ge et al. [34] demonstrated that preoperative hypoalbuminemia is a powerful, independent risk factor for postoperative complications, notably proving that this risk persists even in patients presenting with a completely normal Body Mass Index (BMI). This emphasizes that normal weight can mask underlying severe malnourishment [34].

Expanding beyond traditional serum markers, recent studies leverage advanced cross-sectional imaging to evaluate morphometric features like sarcopenia and myosteatosis. Nagayoshi et al. [28] highlighted the profound impact of a sarcopenic state—defined by a low skeletal muscle mass index (SMI) calculated via CT imaging—as a highly sensitive indicator and independent predictor of postoperative complications [28]. Similarly, Donnelly et al. [24] explored the broader spectrum of body composition, revealing that preoperative myosteatosis (characterized by high intramuscular adipose tissue accumulation) significantly correlates with overall increased postoperative morbidity and higher cumulative complication scores. Together, these studies argue for a shift toward detailed, objective morphometric profiling over basic anthropometric measurements like BMI during preoperative staging [24]. Moving beyond isolated static laboratory values, Müller et al. [42] introduced the prognostic utility of “delta albumin”, proving that the proportional dynamic drop between preoperative and postoperative albumin levels is a far superior predictor of complications following laparoscopic intestinal resection than standard baseline albumin values alone [42].

### 3.4. Optimizing Pharmacology and Surgical Therapy

The impact of preoperative medications on surgical outcomes is an area of intense scrutiny. Corticosteroids remain the most consistent pharmacological predictor of postoperative complications, with Kawamoto et al. [22] identifying steroid coverage as an independent predictor of IASC [22]. Conversely, the landmark PUCCINI study (Cohen et al., [30])—the greatest prospective effort on the subject—concluded that preoperative anti-TNF exposure is safe and not associated with increased risk of postoperative infection or surgical site infection (OR = 1.050), regardless of serum drug concentration. In a total of 947 patients, the infection and SSI rates were similar in patients who were exposed to anti-TNF and those unexposed [30].

For newer agents, Li et al. [26] conducted a meta-analysis of 3225 patients over 9 studies and found that preoperative Ustekinumab exposure does not increase overall (OR = 0.84), infectious, or non-infectious complications compared to anti-TNFs or no biological therapy. While newer small molecules like Upadacitinib have shown safety in phase 3 trials for patients with prior surgery histories, their specific impact on immediate postoperative healing is still being characterized [26].

Regarding surgical technique, Gklavas et al. [36] emphasized in a single-center retrospective study containing 153 patients that surgical history is a fixed risk factor, where previous resection independently correlates with both overall complications (OR = 3.90) and IASC (OR = 4.56). In addition, preoperative anti-TNF agents or other immunosuppressants administered prior to surgery were not a risk factor for POC or IASC. Moreover, patients with longer disease duration (over 10 years), previous intestinal resections, and perianal disease presented with significantly higher rates of both POC and IASC [36]. When analyzing the risk of postoperative anastomotic leaks, Golda et al. [39] highlighted, over a cohort of 470 patients, that preoperative albumin (*p* = 0.004), smoking habits (*p* = 0.005) and perioperative blood transfusion (*p* = 0.038) were all risk factors for anastomotic leak. In addition, anastomotic reinforcement through manual suture oversewing resulted as a protective independent factor [39]. Ultimately, these consistent themes are consolidated in the systematic review and meta-analysis by Losinska et al. [18]. By synthesizing international data pools, their work confirms that modifiable preoperative risk domains, predominantly systemic inflammatory markers like elevated CRP and severe nutritional depletion parameters like hypoalbuminemia, remain the most reliable, ubiquitous predictors of short-term adverse surgical outcomes in Crohn’s disease worldwide. In addition, previous surgery remains one of the most robust risk factors for postoperative complications [18].

Furthermore, in a cohort of 147 patients at a single-center institution, Atasoy et al. [44] highlighted that fistulizing Crohn’s disease is associated with postoperative complications. In addition, while multiple factors contribute, fistulizing disease behavior remains the most consistent single predictor of early postoperative complications in clinical experience [44]. In a comprehensive meta-analysis evaluating 1111 patients, Brennan et al. [43] established that aggressive preoperative nutritional support profoundly reduces overall postoperative risk, dropping complication rates from 61.3% in standard care cohorts to just 20.0%. Specifically, preoperative enteral nutrition (EN) was shown to be vastly superior to standard care, while total parenteral nutrition (TPN) demonstrated a helpful but statistically non-significant positive trend [43].

Sacchetti et al. [29], who presented a 10-year institutional experience of 255 patients undergoing resection, reported an overall baseline postoperative complication rate of 35.7%, identifying active preoperative corticosteroid usage alongside malnutrition as the primary drivers of severe Clavien–Dindo complications and intra-abdominal abscesses [29].

While advanced biologic agents have largely shifted toward a favorable safety profile, preoperative corticosteroid use remains strongly correlated with an elevated risk of overall morbidity and Intra-Abdominal Septic Complications (IASCs), such as anastomotic leakage, deep pelvic abscesses, and wound dehiscence. This detrimental relationship is primarily dose- and duration-dependent. In contemporary clinical cohorts, a threshold of more than 20 mg of prednisone equivalent per day, sustained for a period exceeding two weeks prior to the operative date, serves as a major independent predictor of surgical site infections (SSIs) and impaired fascial healing. The biological mechanism driving this vulnerability is rooted in the systemic immunosuppressive and anti-prohibitive actions of systemic steroids. Corticosteroids inhibit the initial inflammatory cascade required for physiological wound healing by suppressing macrophage migration, decreasing collagen synthesis, and disrupting microvascular proliferation at the nascent bowel anastomosis. Consequently, the surgical field is left structurally weakened and highly susceptible to local bacterial translocation. Crucially, contemporary clinical data emphasize that the risks associated with active, high-dose corticosteroid therapy cannot be mitigated simply by adjusting the surgical technique itself. Rather than abruptly discontinuing these medications—which introduces an unacceptable risk of secondary acute disease flare or adrenal crisis—the evidence strongly advocates for aggressive, multidisciplinary preoperative management. This includes implementing strict, accelerated steroid-tapering protocols in the weeks leading up to elective resections, ideally stabilizing the patient at the lowest possible maintenance dose or transitioning them completely off steroids through the temporary use of exclusive enteral nutrition optimization.

### 3.5. The Importance of Machine Learning in Risk Stratification

The integration of multiple clinical and biological data points into a single predictive model is a complex task, leading to the rise of machine learning (ML) to navigate non-linear relationships. Nardone et al. [23] underscored the potential of ML to advance perioperative optimization, advocating for the transition from traditional research methods to advanced tools grounded in artificial intelligence and computer simulation. They noted that current medical strategies have delayed surgical needs but altered presentation phenotypes, necessitating more robust stratification tools to manage the high incidence of postoperative complications [23].

A cornerstone of this technological shift is the work by Wang et al. [25], who developed models to forecast major complications (Clavien–Dindo < III) such as anastomotic fistula, intra-abdominal sepsis, and intestinal obstruction. Their study demonstrated that a Random Forest (RF) model (AUC = 0.965) significantly outperformed conventional logistic regression (AUC = 0.916). Nardone et al. [23] emphasized that these ML models consistently identify a preoperative CD activity index (CDAI) < 220, diminished serum albumin, and extended operative duration as the most critical variables for risk assessment [25].

Other recent models have expanded the utility of ML into specific surgical decisions. Wang et al. [25] introduced an RF model designed to predict the need for temporary stoma formation after intestinal resection, achieving an AUC of 0.780 in their validation cohort and using the SHapley Additive exPlanation (SHAP) method to interpret the relative importance of clinical predictors.

Furthermore, Neel et al. [57] utilized the NSQIP-IBD database (N = 4256) to model postoperative sepsis, identifying alkaline phosphatase and operative duration as positive predictors, while finding that biologic use within 60 days preoperatively was inversely associated with sepsis risk (AUC = 0.775 for CD) [57]. Despite these advancements, Chen et al. [58] cautioned in their systematic review that many existing models still face a high risk of bias in their analysis domains, highlighting the need for future models to integrate objective assessments of muscle mass and function (sarcopenia) to improve accuracy [58].

### 3.6. Further Directions

The synthesis of evidence over the last decade regarding the risk factors as well as the potential predictive biomarkers in developing postoperative complications in Crohn’s disease surgery emphasizes the synergistic nature of the preoperative risk factors. While individual biomarkers such as CRP or albumin remain significant, their predictive power is enhanced when used in combination with morphological factors such as the sarcopenic index. Sarcopenia has emerged as a superior metric to BMI, with multiple studies highlighting its importance in determining the frailty index of a patient, some studies indicating up to 5 times higher risk of developing postoperative infectious complications. Thus, developing preoperative stratification risks or scores is necessary in order to further individualize the patient’s treatment.

In addition, the necessity of developing proper definitions and validated thresholds for postoperative complications as well as postoperative recurrence should be mandatory to ensure clinical reproducibility as well as a standard of reporting across studies. Future prognostic frameworks should focus on dynamic monitoring instead of a retrospective reactive snapshot to further individualize a patient’s treatment. Finally, the radiological assessment of sarcopenia should be standardized and further implemented into routine clinical perioperative assessment for a more tailored selection of patients.

Although machine learning currently presents with a high-risk of bias, developing multiple studies can help refine AI-driven tools to reach and overcome the current gold standards in endoscopy and clinical assessment of patients. Furthermore, the study of multiomics can reveal novel biomarkers, especially in inflammatory bowel disease, that may highlight subclinical progression and help develop risk stratification strategies in predicting postoperative complications.

**Figure 2:**
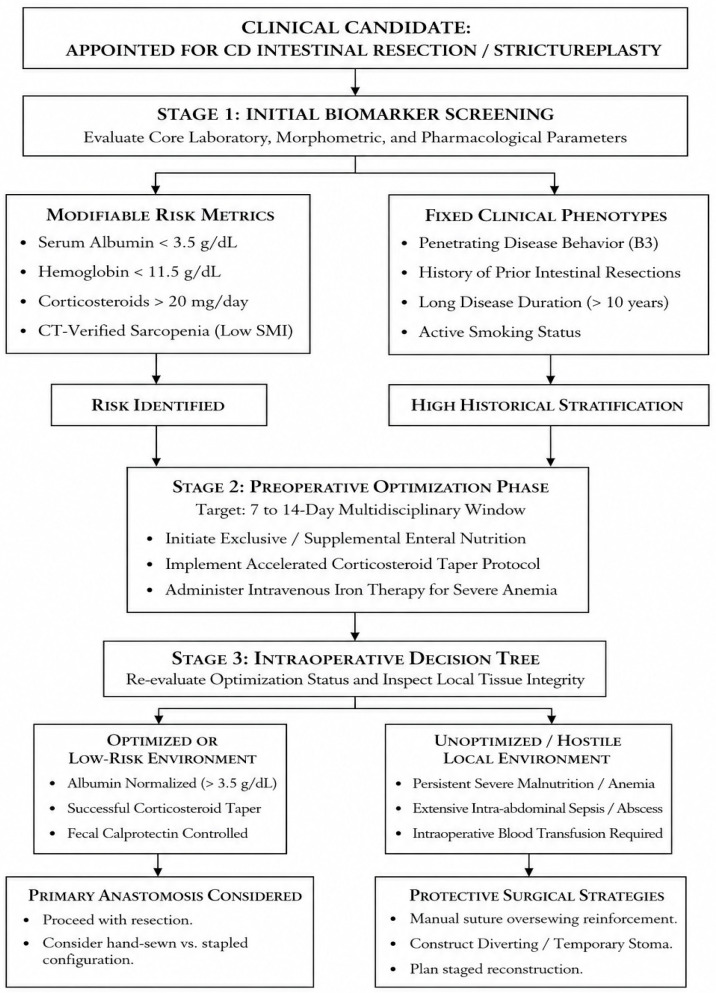
Proposed algorithm for preoperative and intraoperative management.

While non-modifiable features like disease phenotype and stricturing/penetrating behavior establish baseline surgical difficulty, routine modifiable biomarkers dictate immediate postoperative outcomes. Patients demonstrating a triad of hypoalbuminemia (<3.5 g/dL), anemia (<11.5 g/dL) and high-dose corticosteroid use should be selectively funneled into a mandatory 7–14-day preoperative optimization pathway featuring exclusive or supplementary enteral nutrition and strict steroid tapers. This may be clinically relevant as it opens new avenues in preoperative decision-making as well as intraoperative risk stratification and strategy. To successfully translate these systematic insights into reproducible surgical outcomes, we propose a standardized, three-stage clinical optimization pathway structured around objective biomarker thresholds (Figure 2). The foundation of this framework relies on early screening at least two to four weeks prior to scheduled elective surgery. By pairing traditional biochemical metrics with morphometric data, clinicians can differentiate between fixed historical risks and active, modifiable liabilities.

### 3.7. Strengths and Limitations

This review presents a series of key strengths that contribute to the understanding of risk management in postoperative complications in Crohn’s Disease. While maintaining a contemporary framework, it captures the impact of modern biological techniques, as well as postoperative outcomes in a relationship with various disease patterns. In addition, it synthesizes diverse data points ranging from biochemical markers to a multiomics and machine learning approach, which are supported by a large cohort of over 20,000 cases, while maintaining a tempered approach. While previous systematic reviews have extensively evaluated standard biochemical indices such as serum albumin or C-reactive protein in isolation, they often lack integration with contemporary perioperative advancements. This review provides a novel, multi-dimensional synthesis by evaluating how traditional biomarkers intersect with (1) advanced radiological body composition analysis (analytic morphomics/sarcopenia), (2) the changing landscape of newer biologic therapies (e.g., Ustekinumab), and (3) emergent artificial intelligence data structures. By assessing these components through the standardized GRADE and NOS quality assessment frameworks, this study offers a unique, updated paradigm for risk stratification within the modern era of personalized IBD surgery.

However, it has a certain number of drawbacks. Its primary limitation remains the heterogeneity of data, due to variations in study designs, patient population, and an inadequate definition of complications, which prompted to perform a qualitative instead of quantitative analysis. Furthermore, several studies were limited to single-center cohorts, which may further impact the findings, especially in a multifactorial disease such as Crohn’s. There is also an inherent model bias due to the advent of machine learning. Furthermore, the limitation to only English literature as well as non-pediatric literature may omit relevant data regarding specific biomarkers.

## 4. Conclusions

There have been some significant technological advancements in the last decade in terms of machine learning and multiomics. While clinical phenotypes may establish baseline risk, the baseline markers such as albumin, CRP and sarcopenia remain the most critical predictors of postoperative morbidity in Crohn’s disease surgery. Malnutrition and anemia remain the most consistent predictors across cohorts, with preoperative nutritional support limiting this risk. Some hematological markers, notably NLR, can provide an adequate risk assessment of developing postoperative complications; however, due to contradictory evidence, further studies are required to sustain this affirmation. Although machine learning models may provide a robust framework for personalized risk assessment, there is still a significant risk of bias due to missing data or lack of external validation. Further studies are required to emphasize its importance and improve its validity. While non-modifiable features like disease phenotype and stricturing/penetrating behavior establish baseline surgical difficulty, routine modifiable biomarkers dictate immediate postoperative outcomes. Clinical practices must shift from evaluating singular markers to a multi-parametric optimization protocol.

## Figures and Tables

**Table 1:** Selected articles for the review process.

Study (Author, Year)	Article Title	Study Design	Sample Size (*n*)	Key Findings/Outcomes
Losinska et al. [18]	Risk factors for postoperative complications in Crohn’s disease: A systematic review and meta-analysis	Systematic Review and Meta-Analysis		Aggregated data highlights that modifiable preoperative risk domains—specifically systemic inflammatory markers (elevated CRP) and nutritional depletion parameters (hypoalbuminemia)—serve as the strongest consistent predictors of severe short-term adverse surgical outcomes across international cohorts.
Teixeira et al. [19]	Thirty-day postoperative outcomes and predictive factors in Crohn’s disease patients undergoing intestinal resection	Retrospective Multicenter	76	Elevated preoperative CRP was the only independent predictor of adverse outcomes
Naveh et al. [20]	Correlation Between Postoperative Complications and Disease Recurrence	Retrospective Cohort	242	Preoperative FCP only factor influencing complication severity (*p* = 0.05).
Saleh et al. [21]	Evaluating the role of sarcopenia in adverse clinical outcomes for CD	Systematic Review	2334	Sarcopenia increases risk of hospitalization (OR = 1.87) and abscess (OR = 5.03).
Kawamoto et al. [22]	Neutrophil-to-Lymphocyte Ratio as a Biomarker for Postoperative Complications in Crohn’s Disease	Retrospective Observational	206	Preoperative NLR\geq 3.98 is an independent predictor of IASC (OR = 3.43).
Nardone et al. [23]	Advancing perioperative optimization in Crohn’s disease surgery with machine learning predictions	Editorial/Review of ML model	259	ML models identified preoperative CD activity index ≥ 220, low albumin, and long operation duration as critical risk factors for complications.
Donnelly et al. [24]	Obesity, Sarcopenia and Myosteatosis: Impact on Clinical Outcomes in the Operative Management of Crohn’s Disease	Consecutive Retrospective Cohort	124	Preoperative intramuscular adipose tissue (IMAT)—a key marker of myosteatosis—is independently associated with overall increased postoperative morbidity (OR = 1.08, *p* = 0.037) and a higher comprehensive complications index. Conversely, increased adiposity (visceral fat) predicts specific focal risks like venous thromboembolism rather than overall morbidity.
Wang et al. [25]	Predicting short-term major postoperative complications in intestinal resection for Crohn’s disease: A machine learning-based study	Retrospective Cohort Study	259	Low albumin and high CDAI are critical variables for predicting major postoperative complications.
Li et al. [26]	Systematic Review and Meta-Analysis: Association between Preoperative Ustekinumab and Surgical Complications in Crohn’s Disease Patients	Systematic Review	3225	In general, compared with other biological agents, preoperative use of UST in the treatment of CD patients is usually safe and does not increase surgical complications.
Jiang et al. [27]	Role of perioperative nutritional status and enteral nutrition in predicting and preventing postoperative complications in patients with Crohn’s disease	Retrospective Cohort Study	98	Preoperative EN significantly reduced POC rates and LOHS; malnutrition linked to prolonged ileus.
Nagayoshi et al. [28]	Strong impact of sarcopenic state defined by skeletal muscle mass index on postoperative complications in Crohn’s disease patients	Retrospective Cohort Study	137	Skeletal muscle mass index (SMI < 38.9/m^2^) calculated via single-slice CT is an independent predictor and highly useful nutritional indicator for postoperative complications (OR = 2.85, *p* = 0.03), along with low serum albumin.
Sacchetti et al. [29]	Early and late outcomes of a series of 255 patients with Crohn’s disease who underwent resection: 10 years of experience at a single referral center	Retrospective Study	255	Overall postoperative complication rate reached 35.7%. Both corticosteroid use up to 7 days before surgery and active malnutrition (low BMI or severe hypoalbuminemia) significantly increase the risk of developing Clavien–Dindo > 1 complications and postoperative intra-abdominal abscesses.
Cohen et al. [30]	Prospective Cohort Study to Investigate the Safety of Preoperative Tumor Necrosis Factor Inhibitor Exposure in Patients With Inflammatory Bowel Disease Undergoing Intra-abdominal Surgery	Prospective Multicenter Cohort	947	Preoperative TNFi exposure was not associated with postoperative infectious complications in a large prospective multicenter cohort.
Mullin et al. [31]	Inflammatory markers may predict postoperative complications and recurrence in Crohn’s disease patients undergoing gastrointestinal surgery	Retrospective Single-Institution Study	81	Higher preoperative NLR and PLR were associated with major postoperative complications and reoperation in elective patients.
Zhao et al. [32]	Postoperative Ratio of C-Reactive Protein to Albumin as a Predictive Marker in Patients with Crohn’s Disease Undergoing Bowel Resection	Retrospective Cohort Study	534	Postoperative C-reactive protein-to-albumin ratio (CAR) is a strong independent risk factor for complications (OR = 13.200, *p* < 0.001). Using a cutoff threshold of 3.25, a high CAR more accurately predicts complications, longer hospital stays, and surgical site infections than absolute postoperative day 3 CRP values alone.
Zhang et al. [33]	Prevalence of Sarcopenia and Its Effect on Postoperative Complications in Patients with Crohn’s Disease	Retrospective Analysis	124	Sarcopenia is an independent risk factor for major complications (OR = 3.97).
Ge et al. [34]	Preoperative hypoalbuminemia is an independent risk factor for postoperative complications in Crohn’s disease patients with normal BMI: A cohort study	Retrospective Observational Study	315	Low preoperative albumin level (OR = 2.991, *p* = 0.013) is a significant independent risk factor for postoperative complications in Crohn’s disease patients, even when they present with a normal BMI.
Dong et al. [35]	Prognostic significance of the Controlling Nutritional Status (CONUT) score in predicting postoperative complications in patients with Crohn’s disease	Retrospective Database Cohort	202	Preoperative CONUT scores higher than 3.5 identify high-risk malnutrition states and function as a potent independent risk factor for complications (OR = 3.507, *p* = 0.003). The composite immune-nutritional score serves as a better clinical predictor of adverse surgical outcomes than isolated absolute metrics like albumin or the prognostic nutritional index (PNI).
Gklavas et al. [36]	Risk factors for postoperative complications after elective ileocolic resection for Crohn’s disease: a retrospective study	Retrospective single-center	153	Previous resection independently correlated with overall POC (OR = 3.90) and IASC (OR = 4.56).
Ghoneima et al. [37]	High risk of septic complications following surgery for Crohn’s disease in patients with preoperative anemia, hypoalbuminemia and high CRP	Retrospective Observational Study	79	Patients with ≥2 abnormal values (low Hb, low Alb, high CRP) had a 30.7% rate of septic complications.
Nguyen et al. [38]	Hypoalbuminaemia and Postoperative Outcomes in Inflammatory Bowel Disease: the NSQIP Surgical Cohort	Large-scale Surgical Cohort (NSQIP)	6082	Severe hypoalbuminemia (<3.0 g/dL) is strongly associated with 30-day mortality and higher infectious complication rates.
Golda et al. [39]	Risk factors for ileocolic anastomosis dehiscence; a cohort study	Retrospective Single-Institution Cohort	470	Low albumin, smoking, and blood transfusion are risk factors for leaks; suture oversewing reinforcement is protective.
Hu et al. [40]	Incidence and risk factors for incisional SSI in patients with CD undergoing bowel resection	Retrospective analysis	159	Independent predictors for SSI were anemia, elevated neutrophil percentage, and intraoperative fistula.
Argeny et al. [41]	Prognostic value of preoperative neutrophil-to-lymphocyte ratio in Crohn’s disease	Retrospective Cohort Study	373	Elevated preoperative neutrophil-to-lymphocyte ratio in symptomatic Crohn’s disease is not predictive for complications. However, neutrophil-to-lymphocyte-ratio showed a significant correlation with specific disease phenotypes.
Müller et al. [42]	Delta albumin is a better prognostic marker for complications following laparoscopic intestinal resection for Crohn’s disease than albumin alone—A retrospective cohort study	Retrospective Cohort Study	182	The proportional dynamic decrease in perioperative albumin (Delta albumin) is significantly higher in patients experiencing surgical complications. Utilizing a cutoff of 24.27%, Delta albumin acts as an independent prognostic marker for an eventful postoperative course, whereas absolute baseline preoperative or postoperative albumin values taken alone do not significantly correlate with complications.
Brennan et al. [43]	Does preoperative enteral or parenteral nutrition reduce postoperative complications in Crohn’s disease patients: a meta-analysis	Systematic Review and Meta-Analysis	1111	Preoperative nutritional supplementation profoundly mitigates postoperative risk (20.0% complication rate with nutritional support vs. 61.3% with standard care). Preoperative enteral nutrition (EN) is highly effective and significantly superior to standard care (OR = 0.09, *p* < 0.001), while total parenteral nutrition (TPN) exhibits a positive but statistically non-significant trend.
Atasoy et al. [44]	Predictive parameters of early postoperative complications in CD: Single team experience	Retrospective analysis	147	Fistulizing disease behavior was the only significant predictor of early postoperative complications.
Stidham et al. [45]	Body Fat Composition Assessment Using Analytic Morphomics Predicts Infectious Complications After Bowel Resection in Crohn’s Disease	Retrospective Cohort Study	269	Subcutaneous-to-visceral fat distribution, surgical urgency, and low hemoglobin were significant predictors of infectious complications.

**Table 2:** NOS scores.

Study (Author, Year)	Sample Size (*n*)	Group/Conclusion Point	NOS
**Inflammatory Markers**			
Kawamoto et al. [22]	206	NLR > 3.89 predicts IASC (OR = 3.43).	7/9 (Good)
Teixeira et al. [19]	76	CRP is the only independent predictor of outcomes.	7/9 (Good)
Ghoneima et al. [37]	79	Synergistic effect of low Hb, Alb, and high CRP.	6/9 (Fair)
Mullin et al. [31]	81	NLR and PLR are associated with reoperation risk.	7/9 (Good)
Zhao et al. [32]	534	CRP to Albumin ratio is a strong predictive factor for postoperative complications	8/9 (Good)
Dong et al. [35]	202	Preoperative scores > 3.5 identify high-malnutrition state	8/9 (Good)
Hu et al. [40]	159	Anemia and intraoperative fistula predict SSI.	7/9 (Good)
Argeny et al. [41]	373	NLR does not predict complications	8/9 (Good)
**Nutrition & Morphomics**			
Saleh et al. [21]	2334	Sarcopenia increases abscess risk 5-fold.	8/9 (Good)
Donnelly et al. [24]	124	IMAT is associated with high postoperative morbidity	7/9 (Good)
Nagayoshi et al. [28]	137	Low SMI is a highly useful nutritional indicator for postoperative complications	8/9 (Good)
Jiang et al. [27]	98	Preoperative EN reduces POC and stay length.	7/9 (Good)
Nguyen et al. [38]	10,913	Hypoalbuminemia predicts 30-day mortality.	8/9 (Good)
Bae et al. [46]	227	Low PNI (<40) increases infectious risk.	7/9 (Good)
Zhang et al. [33]	124	Sarcopenia is an independent complication risk.	7/9 (Good)
Ge et al. [34]	315	Low preoperative albumin predicts postoperative complications even in normal BMI	8/9 (Good)
Müller et al. [42]	373	Delta albumin is significantly higher in patients expecting postoperative complications	7/9 (Good)
Stidham et al. [45]	269	Subcutaneous-to-visceral distribution ratio can predict complications	7/9 (Good)
**Machine Learning (ML)**			
Wang et al. [25]	259	Random Forest model superior to logistic.	7/9 (Good)
Nardone et al. [23]	259	ML enables optimized perioperative stratification.	N/A (Editorial)
**Pharmacology & Surgery**			
Losinska et al. [18]	51	Elevated CRP, previous surgery and hypoalbuminemia remain the strongest risk factors	9/9 (Good)
Golda et al. [39]	470	Smoking and transfusion increase dehiscence.	7/9 (Good)
Atasoy et al. [44]	147	Fistulizing behavior is the primary risk driver.	7/9 (Good)
Li et al. [26]	3225	Ustekinumab is safe for preoperative use.	7/9 (Good)
Cohen et al. [30]	955	PUCCINI: Anti-TNFs do not increase infection.	9/9 (Good)
Sacchetti et al. [29]	947	Corticosteroid up to 7 days prior to surgery and malnutrition are the most important risk factors	7/9 (Good)
Gklavas et al. [36]	153	Previous resection correlated with POC (OR = 3.90)	8/9 (Good)
Brennan et al. [43]	182	Perioperative nutrition profoundly mitigates postoperative risk	9/9 (Good)

**Table 3:** Synthesis and strength of evidence for predictive biomarkers in CD surgery.

Category	Specific Biomarker	Primary Association	Evidence Strength (GRADE)	Clinical Actionability/Threshold
Nutritional/Morphometric	Serum Albumin	Overall complications, deep infection, leak	High (Consistent across NSQIP and cohort data)	<3.5 g/dL; requires 14 days of preoperative enteral nutrition optimization.
	Sarcopenia (Skeletal Muscle Index via CT)	Abscess formation, prolonged hospitalization	Moderate to High	Visualized on routine staging CT; dictates high protein prehabilitation.
Systemic Inflammatory	C-Reactive Protein (CRP)	30-day adverse outcomes, acute surgical flare	High	Elevated baseline requires checking for undrained sepsis before resection.
	NLR/PLR Ratios	Major complications, reintervention, thromboembolism	Moderate (Some conflicting data, e.g., Argeny et al. [41])	NLR > 4.1 or PLR elevation signals systemic hyper-inflammation.
Local Inflammatory	Fecal Calprotectin (FCP)	Severity of complications (Clavien–Dindo), mucosal healing status	Moderate	High levels predict active anastomotic zone inflammation; markers for postoperative recurrence monitoring.
Advanced Tools	Machine Learning (Random Forest)	Non-linear risk modeling (AUC up to 0.96)	Low to Moderate (High risk of bias/lack of external validation)	Not ready for standalone clinical use; requires open-source web-calculators.

## Data Availability

The original contributions presented in this study are included in the article. Further inquiries can be directed to the corresponding author.
